# Aberrantly expressed mRNAs and long non-coding RNAs in patients with invasive ductal breast carcinoma: A pilot study

**DOI:** 10.3892/mmr.2014.2989

**Published:** 2014-11-20

**Authors:** XUEDONG CHEN, JINGYUN YANG, LIYUAN QIAN, TIANZHU CAO

**Affiliations:** 1 Department of Breast and Thyroid Surgery, The Third Xiangya Hospital, Central South University, Changsha, Hunan 410013, P.R. China; 2 Rush Alzheimer’s Disease Center, Rush University Medical Center, Chicago, IL 60612, USA; 3 Department of Neurological Sciences, Rush University Medical Center, Chicago, IL 60612, USA

**Keywords:** mRNA, long non-coding RNA, invasive ductal breast carcinoma, expression

## Abstract

Invasive ductal breast carcinoma (IDBC) is the most prevalent type of invasive breast cancer in females; however, the pathogenesis of IDBC remains to be elucidated. Therefore, the identification of novel markers may enhance current understanding of the initiation and development of IDBC as well as elucidate potential therapeutic targets for effective treatment of IDBC. In the present study, a pilot study was conducted to screen for potential mRNAs and long non-coding (lnc)RNAs that exhibit aberrantly altered expression in patients with IDBC. Fresh breast cancer specimens and normal breast tissues were obtained from three female patients with IDBC aged ≥60 years following a modified radical mastectomy without chemotherapy. Expression levels of 44,244 probes were detected and included in the analysis, of which 22,078 (49.9%) were mRNAs and 22,166 (50.1%) were lncRNAs. Potential marker screening was performed using paired t-tests (criterion 1), false discovery rates (FDR; criterion 2) and sure independence screening procedures based on distance correlations (DC-SIS; criterion 3). The results showed that in IDBC tissues 3,510 probes had a ≥2-fold statistically significant change in expression levels compared to those in the corresponding normal breast tissue (P<0.05); in addition, following FDR analysis, 353 probes were found to have significantly altered expression levels. Furthermore, DC-SIS analysis identified 18 probes (12 mRNA and 6 lncRNAs) with significantly altered expression levels in IDBC tissue; these 18 probes therefore demonstrated significant results in all three criteria. Several of the mRNAs identified have been previously reported to be involved in signal transduction, protein binding, and cancer pathways, and the present study revealed that the majority of their gene products were located in the cytoplasm. Two of the six identified lncRNAs demonstrated a >10-fold decrease in expression levels in IDBC tissues compared to that in the normal breast tissue. However, further studies are required in order to elucidate the biological functions of the identified probes.

## Introduction

Breast cancer is the most prevalent type of malignant cancer in females worldwide, of which invasive ductal breast carcinoma (IDBC), also known as infiltrating ductal carcinoma, accounts for 70–80% of invasive breast cancer cases (1). Previous studies have indicated that IDBC has multiple stages of development, initiating from premalignant hyperplastic breast lesions, which progress to ductal breast cancer *in situ* (DCIS) and then to IDBC (2–4). It was reported that an interim stage, DCIS with microinvasion, may also have an important role in the progression from DCIS to metastatic IDBC (5). This linear multi-step model of human breast cancer progression serves as a starting point for current understanding of breast cancer pathogenesis; however, numerous studies have contradicted this model (4,6). Therefore the complex pathogenesis of IDBC remains to be elucidated.

mRNA conveys the genetic information in DNA into the translation of amino acids. Many studies have reported that the expression of mRNAs was altered in breast cancer tissues (7–9); therefore, mRNA expression may be used to predict the prognosis of patients with IDBC (10). A previous study into the transcriptomic landscape of breast cancer using in-depth mRNA sequencing revealed numerous novel and annotated transcripts in breast cancer tissue; this therefore reflected the limited current understating of mRNAs in the pathogenesis of the disease (8).

Long non-coding RNAs (lncRNA) are non-coding RNAs consisting of >200 nucleotides. lncRNAs were previously considered to be ‘junk DNA’; however, studies have demonstrated that lncRNAs participated in the regulation of protein transcription and epigenetic modification, and were reported to be involved in a variety of developmental processes as well as several diseases (11–13). Only a small number of lncRNAs have been studied extensively; therefore, the function of numerous lncRNAs remains to be elucidated (14). In addition, the identification of novel lncRNAs and exploration of their underlying regulatory mechanisms in the initiation and progression of diseases is essential for a deeper understanding of disease pathogenesis. Previous studies have also demonstrated the altered expression of lncRNAs in breast cancer, including Hox transcript antisense intergenic RNA (HOTAIR) and growth arrest-specific 5 (15–17). However, the biological functions of the majority of lncRNAs in association with IDBC remain to be elucidated. In the present study, a pilot study was conducted to explore novel mRNAs and lncRNAs that exhibit aberrantly altered expression in patients with IDBC, which may therefore potentially be involved in the pathogenesis of IDBC.

## Materials and methods

### Participants

In June, 2013, three female patients aged ≥60 years and diagnosed with IDBC underwent a modified radical mastectomy without chemotherapy at the Department of Breast and Thyroid Surgery of the Third Xiangya Hospital (Changsha, China). The criteria for IDBC diagnosis was as follows: Pathological examination which revealed a tumor with a diameter >2cm and <5cm in the presence of lymph node metastasis. Pathological examinations were performed by experienced clinicians in clinical pathology at the Third Xiangya Hospital. All three participants were diagnosed with stage III IDBC according to the Bloom-Richardson grading system (18). Informed consent was obtained from each participant, and the study was conducted in adherence to the tenets of the Declaration of Helsinki. This study was approved by the ethics committee of the Third Xiangya Hospital of Central South University.

### Resection of breast specimens

Fresh breast cancer specimens and normal breast tissues were obtained from the participants during a modified radical mastectomy, and each surgery was performed by the same experienced surgeon. Normal breast tissue samples were resected from breast glands >5cm distance from the tumor tissue. Following surgery, the breast tissue samples were diagnosed by pathological clinicians, then preserved in liquid nitrogen within 30 min and stored at −80°C.

### RNA microarray and hybridization

Total RNA was extracted using the mirVana RNA Isolation kit (Life Technologies, Grand Island, NY, USA). RNA quality control, labeling and hybridization were performed by Shanghai Biochip Co., Ltd. (Shanghai, China) according to the manufacturer’s instructions of the Agilent microRNA Microarray System 2.4 (Agilent Technologies, Santa Clara, CA, USA). Arrays were then scanned using an Agilent Microarray Scanner (G2505C; Agilent Technologies) and the fluorescence intensities of the labeled samples were normalized according to the median of the total signals on the arrays. Images were captured using Scanner Control Software 7.0 (Agilent Technologies) and signal intensities were analyzed using ArrayVision 6.0 software (Imaging Research, St. Catharines, ON, Canada).

### Target gene analysis

TargetScan Human release 6.0 online software (http://www.targetscan.org/vert_60/) was used to predict microRNA targets as previously described (19,20). The Database for Annotation, Visualization and Integrate Discovery v6.7 (http://david.abcc.ncifcrf.gov/) was then used to annotate the biological functions of predicted targets as previously described (21,22).

### Statistical analysis

All experiments were performed in triplicate. Screening for differentially expressed mRNA or lncRNA was performed using paired t-test (criterion 1), and significance was indicated by a threshold of ≥2-fold change in expression and a corresponding P-value of ≤0.05. False discovery rate (FDR) analysis was used to adjust for multiple testing (criterion 2) and Q≤0.05 was considered to indicate a significant change in expression between groups.

Sure independence screening procedure based on the distance correlation (DC-SIS), was then performed in order to compare the findings (criterion 3) (23). DC-SIS is a novel statistical method for screening important characteristics for ultra-high dimensional data; in addition, DC-SIS does not make any model assumption (e.g. linear model) for the response (e.g. breast cancer or not) and the predictors (e.g. expression of mRNAs or lncRNAs), which therefore makes model misspecification highly unlikely. Sure independence ensures that all important variables may be selected with sufficient sample size, which enables DC-SIS to be a more flexible and reliable for screening important predictors compared to conventional statistical methods such as the t-test. Due to the limited sample size in the present study, a model of size 6[n/log(n)], where n is the sample size and [n/log(n)] is the integer part of n/log(n), was selected in order to reduce the possibility of missing important probes. Statistical analyses were performed using R software (www.R-project.org) and SAS 9.3 (SAS Institute Inc., Cary, NC, USA).

## Results

### Interrogated probes

A total of 44,244 probes, which consisted of 22,078 mRNA and 22,166 lncRNA probes (49.9 and 50.1%, respectively), were interrogated and included in the final analyses.

### Aberrant expression of mRNAs and lncRNAs in IDBC tissue

Paired t-tests located 3,510 probes with statistically significant expression levels changes of ≥2-fold (P≤0.05) in IDBC tissue compared with those of normal breast tissue. A total of 2,090 mRNAs (9.5% of interrogated mRNA probes) demonstrated significant changes, of which 722 (34.5%) showed elevated expression; in addition, 1,420 lncRNAs (6.4% of interrogated lncRNA probes) showed significantly altered expression in IDBC tissue, of which 304 (21.4%) exhibited elevated expression (Table I).

FDR analysis revealed that a total of 353 probes demonstrated significant changes in expression levels in IDBC tissue compared with those of normal breast tissue. Of note, 195 mRNAs (0.9%) showed significant changes, of which 79 (40.5%) exhibited elevated expression; in addition, 158 lncRNAs (0.7%) showed significantly altered expression, of which 37 (23.4%) demonstrated increased expression levels (Table I).

DC-SIS feature screening identified 18 probes which demonstrated significantly altered expression in IDBC tissue compared with that of normal breast tissue. Of these 18 identified probes, 12 were mRNAs (0.05%), three (25%) of which exhibited elevated expression, and six were lncRNAs (0.03%), all of which showed significantly downregulated expression (Table I). These 18 probes were therefore demonstrated to have altered expression levels in IDBC tissues by all the three criteria. Table II provides detailed information regarding these 18 selected probes.

### Functional analysis

**Figure 1:**
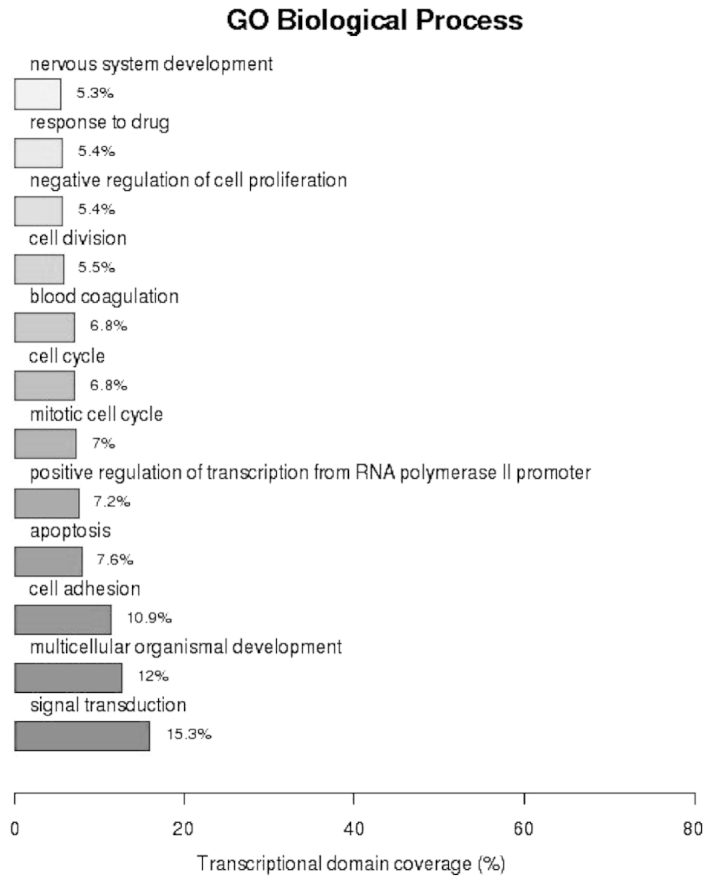
GO analysis of the biological process of the mRNAs identified to be aberrantly expressed in invasive ductal breast carcinoma tissues using paired t-tests following microarray analysis. GO, gene ontology.

**Figure 2:**
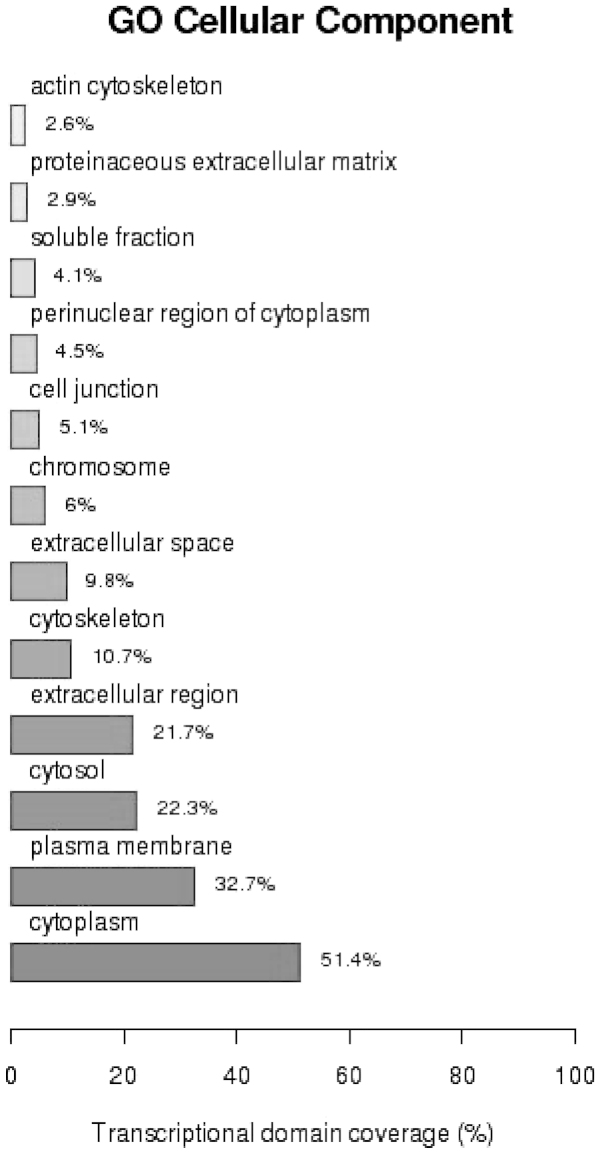
GO analysis of the cellular components of the mRNAs identified to be aberrantly expressed in invasive ductal breast carcinoma tissues using paired t-tests following microarray analysis. GO, gene ontology.

**Figure 3:**
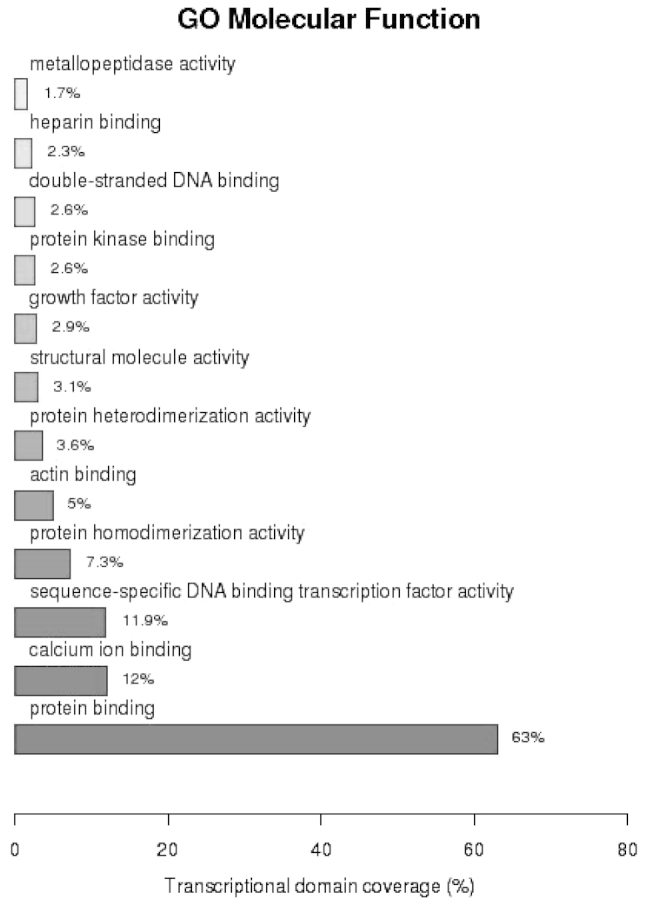
GO analysis of molecular functions of the mRNAs identified to be aberrantly expressed in invasive ductal breast carcinoma tissues using paired t-tests following microarray analysis. GO, gene ontology.

**Figure 4:**
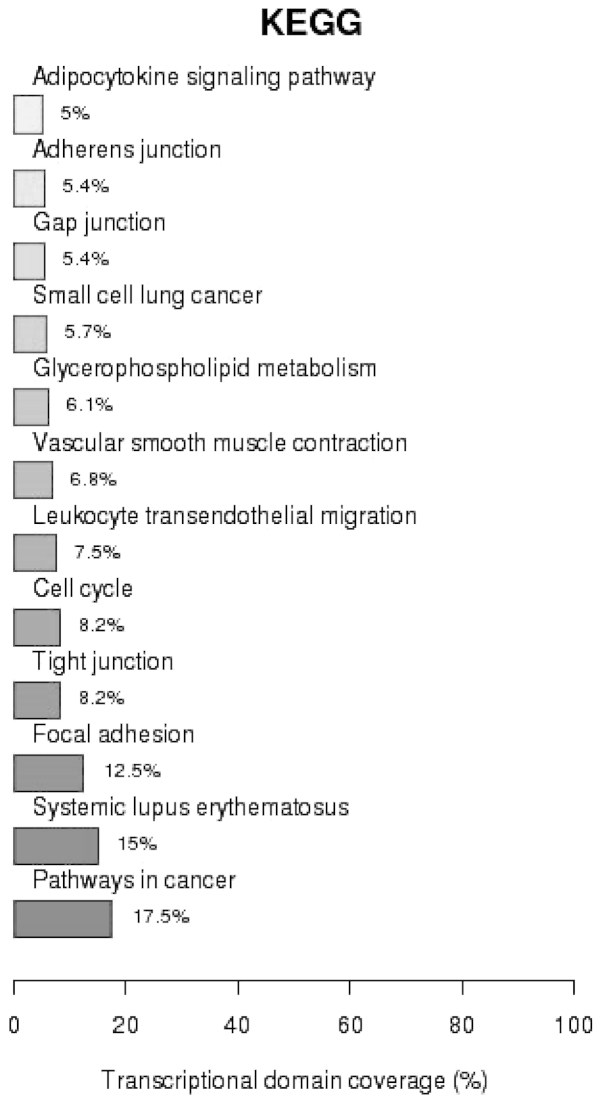
KEGG pathway analysis of the mRNAs identified to be aberrantly expressed in invasive ductal breast carcinoma tissues using paired t-tests following microarray analysis. KEGG, Kyoto Encyclopedia of Genes and Genomes.

Gene ontology (GO) analysis was performed in order to determine the biological functions of genes harboring the 2,090 mRNA probes which were found to be aberrantly expressed in IDBC tissue compared to that of normal breast tissue. The results of the GO analysis of biological functions (Fig. 1) revealed that 15.3% of the genes were involved in signal transduction [enrichment value (P)=1.53×10^−3^], 12% had functions in multi-cellular organism development (P=6.14×10^−10^) and 10.9% were involved in cell adhesion (P=2.01×10^−10^). As shown in Fig. 2 GO analysis of the cellular components of the mRNAs showed that 51.4% of the gene products were located in cytoplasm (P=4.9×10^−6^), 32.7% in the plasma membrane (P=4.24×10^−2^) and 22.3% in the cytosol (P=5.18×10^−5^). GO analysis of molecular function demonstrated that 63% of the genes were involved in protein binding (P=5.82×10^−5^), 12% in calcium ion binding (P=7.27×10^−9^) and 11.9% in sequence-specific DNA binding transcription (P=2.16×10^−2^) (Fig. 3). As shown in Fig. 4, analysis of the Kyoto Encyclopedia of Genes and Genomes (KEGG) pathways revealed that 17.5% of the genes identified were associated with cancer pathways (P=1.37×10^−3^), 15% were involved in systematic lupus erythematosus (SLE; P=6.02×10^−12^) and 12.5% in focal adhesion (P=4.18×10^−4^).

## Discussion

In the present study, mRNA and lncRNA expression levels were detected in the normal and cancerous tissues from three patients with IDBC. Following microarray analysis, numerous aberrantly expressed mRNAs and lncRNAs were located in IDBC samples. The majority of genes which harbored the differentially expressed mRNAs were found to be involved in signal transduction, protein binding and cancer pathways, and their gene products were predominantly located in cytoplasm.

One of the identified mRNAs A_23_P315364 is found in the *CXCL2* gene, located on chromosome 4. C-X-C motif ligand 2 (CXCL2) is a chemokine that is highly expressed in metastases (24). A previous study identified a paracrine network between tumor and stromal cells comprising of CXCL1 and 2, which indicated that lung metastasis was associated with chemotherapy resistance in breast cancer (25). Another mRNA identified in the present study, A_33_P3290338, is found in the *PARP1* gene, which encodes a nuclear enzyme that has an important role in regulating DNA repair (26). One study performed a meta-analysis which showed that *PARP1* mRNA expression was heterogeneous between breast cancer subtypes and was overexpressed in 58% of breast cancers (9); this was concurrent with the results of the present study, which found that *PARP1* expression was elevated in patients with IDBC. In addition, mRNA expression of *PARP1* was associated with high medullary histological grade, tumor size, metastasis-free survival (MFS) and overall survival in patients with breast cancer, and is an independent prognostic factor for MFS (9). However, further studies are required in order to elucidate the exact biological/molecular functions and pathways of mRNAs identified in the present study.

In the present study, 18 probes were identified which were found to have significantly altered expression in IDBC tissue according to all the three criteria. Six of the identified probes were lncRNAs, each of which was reported to be downregulated in the IDBC samples compared with that of the normal breast tissue; however, further studies into the function of these lncRNA are required in order to elucidate the mechanism through which they are involved in the pathogenesis of IDBC, as previous studies are limited. Of note, in the present study, two lncRNAs were identified in IDBC tissue which demonstrated a >10-fold decrease in expression: ENST00000458316 (corresponding probe oebiotech_09186) is located on chromosome 21 and was reported to be expressed at higher levels in breast tissue compare various other tissues in the human body (Illumina Human BodyMap 2.0, ArrayExpress ID, E-MTAB-513; http://www.ebi.ac.uk/arrayexpress); and NR_072979 (corresponding robe oebiotech_22954) is a transcript variant of aldehyde dehydrogenase 1 family, member L1 (*ALDH1L1*). The gene product of *ALDH1L1*,10-formyltetrahydrofolate dehydrogenase (FDH) is a major folate-metabolizing enzyme involved in the regulation of cell proliferation (27). FDH was reported to be ubiquitously downregulated in human tumors (27), the mechanism of which was suggested to proceed via promoter methylation which influenced levels of FDH (28); however, the exact effect of this lncRNA on FDH levels and its subsequent influence on cell proliferations remains to be elucidated.

Previous studies have identified several lncRNAs involved in the pathogenesis, progression and survival of breast cancer. HOTAIR, a widely studied lncRNA located on 12q13.13, is transcribed from the antisense strand of *HOXC12* (29) and serves as an interface between DNA and specific chromatin remodeling. Of note, HOTAIR specifies the pattern of histone modifications on target genes by providing binding surfaces for polycomb repressive complex 2 (PRC2) via its 5′ domain as well as providing binding surface for the lysine-specific demethylase 1A/co-repressor element-1-specific transcription factor (CoREST)/REST complex via its 3′ domain (16). A previous study reported that increased expression levels of HOTAIR in primary breast cancer tumors was a prognostic factor for metastasis and death (15). In the present study, HOTAIR expression in IDBC tissue was not found to be significantly decreased. A previous study also reported that of the 336 tumor samples analyzed, HOTAIR expression was markedly varied in breast cancer tissues and 6.5% had undetectable HOTAIR expression; in addition, no association was found between HOTAIR expression and the clinical or pathologic characteristics of breast cancer (30). Furthermore, patients with higher expression levels of HOTAIR demonstrated a lower risk of relapse and death than those with lower expression of HOTAIR (30). These findings are consistent with the results of the present study; however, further studies are required to validate the findings.

SLE is an autoimmune rheumatic disease which occurs primarily in females. Previous studies reported that females with SLE had a decreased risk of developing breast cancer [odds ratio (OR)=0.76, P=2.49×10^−7^] (31) as well as ductal carcinoma (OR=0.95, P=0.067) (32). However, a study of ten lupus-associated single nucleotide polymorphism (SNPs) found less supportive evidence for the association of these SNPs with breast cancer (33), indicating that epigenetic factors may have contributed to the decreased risk of breast cancer in females with SLE. In the present study, KEGG analysis showed that there was an enrichment of genes involved in SLE, indicating that epigenetic factors may be involved in influencing the risk of breast cancer. Of note, the mRNA ENST00000330452 for *PRKCD* exhibited a 1.2-fold increase in expression in IDBC tissues; mutations in *PRKCD* were previously reported to result in the reduced expression and activation of protein kinase C, which in turn may lead to increased B cell proliferation and susceptibility to SLE (34). The results of the present study were consistent with the reported decreased risk of breast cancer in patients with SLE; however, further studies are required in order to elucidate the mechanism underlying the reduced risk of breast cancer, which may further current understanding of its etiology.

The limitations of the present study are due to its small sample size, which therefore prevented conclusive results being reached. However, the significant probes identified by the three criteria represented potential markers and require further investigation. In addition, all three participants had stage III IDBC, which therefore prevented the comparison of aberrant RNA expression among the different stages of IDBC. Furthermore, due to the cross-sectional nature of the present study, the pattern of changes throughout the development of IDBC was not analyzed.

In conclusion, microarray analysis was performed in the present study in order to screen for mRNAs and lncRNAs exhibiting aberrantly altered expression in patients with IDBC compared to that in the normal breast tissue of the same patients. A total of 18 mRNAs and lncRNAs showing significant changes in expression were identified, of which six were lncRNAs and 12 were mRNAs. Functional analysis of the identified mRNA probes demonstrated that they were located in genes involved in various biological functions, including signal transduction and protein binding as well as cancer pathways; however, the functions of the six identified lncRNAs remains to be elucidated. Therefore, further studies are required in order to determine the functions of the identified lncRNAs as well as to validate the results of the present study using larger sample sizes.

## Figures and Tables

**Table I:** Differential expression of mRNAs and lncRNAs in invasive ductal breast carcinoma samples compared to that of normal breast tissue from the same patients.

	mRNA			lncRNA		
		
Statistical criteria	Increased	Decreased	Total	Increased	Decreased	Total
Paired t-test (n, %)^a^	722 (34.5)	1368 (65.5)	2090	304 (21.4)	1116 (78.6)	1420
FDR	79 (40.5)	116 (59.5)	195	37 (76.6)	121 (76.6)	158
DC-SIS	3 (25.0)	9 (75.0)	12	0 (0)	6 (100)	6

a Significance was indicated by ≥2-fold change in expression and a corresponding P-value of ≤0.05.

lncRNA, long non-coding RNA; FDR, false discovery rate; DC-SIS, sure independence screening based on the distance correlation.

**Table II:** Detailed information for the 18 probes identified using DC-SIS.

Probe	Chr	Type	Gene	IDBC	Normal	P-value	Q	Rank
oebiotech_26202	15	lncRNA	NA	7.51	9.14	1.93×10^−6^	1.93×10^−2^	1
oebiotech_08007	17	lncRNA	NA	7.89	8.08	2.57×10^−6^	1.93×10^−2^	2
A_33_P3371999	5	mRNA	*TPPP*	2.58	5.40	4.25×10^−6^	1.93×10^−2^	3
A_23_P144054	3	mRNA	*PRKCD*	10.00	8.84	4.96×10^−6^	1.93×10^−2^	4
A_33_P3320197	2	mRNA	*FAM150B*	2.41	5.70	6.80×10^−6^	2.31×10^−2^	5
oebiotech_09186	21	lncRNA	NA	2.41	6.18	4.94×10^−6^	1.93×10^−2^	6
A_23_P315364	4	mRNA	*CXCL2*	2.59	9.56	4.47×10^−6^	1.93×10^−2^	7
A_21_P0011386	15	mRNA	*LOC100505679*	8.19	9.68	3.64×10^−6^	1.93×10^−2^	8
A_33_P3419691	7	lncRNA	*GATS*	7.16	7.93	9.41×10^−6^	2.84×10^−2^	9
A_33_P3372426	21	mRNA	*ADAMTS5*	2.38	5.46	1.11×10^−5^	2.97×10^−2^	10
oebiotech_22954	3	lncRNA	NA	2.38	5.86	2.51×10^−5^	3.14×10^−2^	11
A_33_P3300262	2	mRNA	*VIT*	2.46	5.85	2.50×10^−5^	3.14×10^−2^	12
oebiotech_19472	3	lncRNA	NA	7.34	9.07	3.35×10^−5^	3.14×10^−2^	13
A_33_P3290338	1	mRNA	*PARP1*	10.01	8.63	2.30×10^−5^	3.14×10^−2^	14
A_33_P3360087	7	mRNA	*BBS9*	8.09	8.82	2.37×10^−5^	3.14×10^−2^	15
A_24_P393958	1	mRNA	*DNAJB4*	6.73	8.20	1.20×10^−5^	2.97×10^−2^	16
A_24_P189533	11	mRNA	*ENDOD1*	7.73	8.52	2.30×10^−5^	3.14×10^−2^	17
A_33_P3325914	6	mRNA	*TAPBP*	13.16	12.19	3.90×10^−5^	3.21×10^−2^	18

DC-SIS, sure independence screening based on the distance correlation; Chr, chromosome where the probe is located; NA, not applicable; normal, normalized mean expression in normal breast tissues; IDBC, normalized mean expression in invasive ductal breast carcinoma tissue; Q, Q-value obtained using the false discovery rate; rank, rank of importance obtained using DC-SIS; lncRNA, long non-coding RNA.
